# A review of the biology of Australian halophilic anostracans (Branchiopoda: Anostraca)

**DOI:** 10.1186/2241-5793-21-21

**Published:** 2014-11-24

**Authors:** Brian Timms

**Affiliations:** Australian Museum, 6-9 College St, Sydney, NSW 2000 Australia; Centre for Ecosystem Science, School of Biology, Earth and Environmental Sciences, University of New South Wales, Sydney, 2052 Australia

**Keywords:** *Artemia*, *Branchinella*, *Parartemia*, Taxonomy, Ecology, Physiology, Conservation

## Abstract

Australia has two species of *Artemia: A. franciscana* introduced to salt works and apparently not spreading, and parthenogenetic *Artemia* presently spreading widely through southwestern Australia. In addition, and unique to Australia, there are 18 described species of *Parartemia* in hypersaline lakes. All *Parartemia* use a lock and key mechanism in amplexus and hence have distinctive antennal-head features in males and thoracic modifications in females. Various factors, including climatic fluctuations and isolation, contribute to a far higher diversity in the southwest of the continent. There are few congeneric occurrences of *Parartemia* possibly due to their consuming largely uniform allochthonous organic matter rather than multisized planktonic algae. In *P. zietziana* there are 2–3 cohorts a year each persisting 3–9 months. Up to 80% of assimilation is used in respiration and at times energy balance is negative, which accounts for its continuous mortality, inconsistent growth rates and unpredictable recruitment. Many species are as osmotically competent as *Artemia*, but are at a disadvantage in hypersaline waters >250 g L^-1^ as they lack the haemoglobin of *Artemia. Parartemia acidiphila* and *P. contracta* live in markedly acid waters to pH 3.5, where dissolved carbonate and bicarbonate are unavailable, and hence they must have evolved an additional proton pump to produce ATP from endogenous CO_2_. Occurrences of some species have been severely curtailed by lake salinisation (which includes acidification and changes in hydroperiod), so that their continued existence is in doubt. A few species of the otherwise freshwater *Branchinella* occur in mainly hyposaline waters.

## Introduction

Truly halophilic anostracans occur in two monogeneric families in the Artemiina, a major subdivision of the Anostraca. The Artemiidae contains *Artemia*, with six bisexual species and several parthenogenetic strains worldwide [1], whereas the Parartemiidae encompasses *Parartemia,* an Australian endemic genus of 18 described species [2]. Australia has a plethora of anostracan species living in inland saline waters, more than any other continent, and besides these, there are many *Branchinella*, mainly in hyposaline waters [3]. Naturally occurring *Artemia* exist in a few isolated lakes in coastal Western Australia near Perth [4], which may represent a parthenogenetic strain of one of the widespread Eurasian species [5]. In addition, the New World *Artemia franciscana* Kellogg, 1906 has been introduced to Qld, SA and WA. These Australian halophilic species largely fill the niche of *Artemia* and *Phallocryptus* on other continents.

Realisation of the diversity of halophilic Anostraca is only recent, with most of the ecology of *Parartemia* being researched in Victoria on *P. zietziana* in the 1970s and most of taxonomy in the 1940s and since 2000. There has been no integrated account of *Artemia* and *Parartemia* in Australia since Williams & Geddes [6] and the situation now is quite different, especially with improved knowledge of *Branchinella*’s foray into saline waters and of conservation issues. It is the purpose of this review to bring the scattered published and grey literature together to provide an integrated ecology of brine shrimps in Australia. It will also reveal some major knowledge gaps.

## Review

### *Artemia*in Australia

Over a century ago, three endemic species of *Artemia* were described from Australian waters. These were *A. proxima* King, 1855, *A. westraliensis* Sayce, 1903 (*nomina nuda*, see [2]) and *A. australis* Sayce, 1903 (*nomen dubium*, see [2]). The descriptions are inadequate and no material exists for *A. proxima*, which is surmised to be *Artemia*, but introduced on salt making equipment. The other two are a few distorted females in museums: the first is a *Branchinella* female, most of which are indistinguishable from one another, and the second is a *Parartemia*[7, 8].

**Figure 1:**
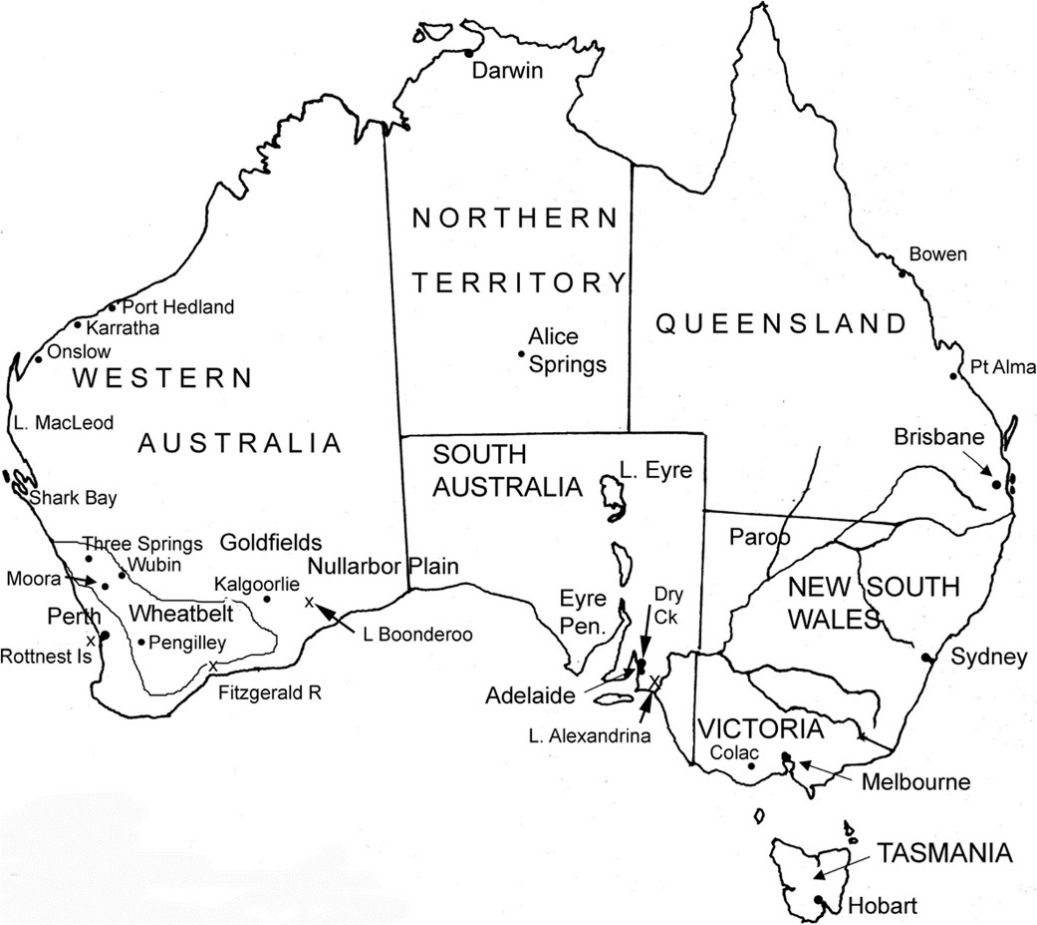
**Map of Australia showing most of the places mentioned in the text.**

*Artemia franciscana* has been introduced to a number of salt works such as at Bowen and Port Alma in Qld, Dry Creek near Adelaide in SA (where it has replaced parthenogenetic *Artemia*; P. Coleman, pers. comm.), Port Hedland and Karrata in WA (M. Coleman, pers. comm.) (Figure 1). Parthenogenetic *Artemia* occurs in salt works at Onslow, Lake McLeod, Shark Bay and also in an industrial works at Three Springs, WA, often colonising of their own accord (M. Coleman & B. Datson, pers. comm.) (Figure 1). This species occurs naturally in salt lakes on Rottnest Is off Perth and in some coastal lakes south of Perth [4] but is also presently spreading across the WA Wheatbelt to sites including Coomberdale near Moora, northeast of Wubin, east of Pengilley, and Fitzgerald River (G. Janicke, pers. comm. & author’s unpublished data) into secondarily salinised lakes and river pools where the environment is now more suited to them than to native *Parartemia*. It also occurs in the remote and highly episodic Lake Boonderoo on the Nullarbor Plain where there are no records of *Parartemia* ever being present, but this may be due to this lake’s remoteness (J. Lane, pers. comm.) (Figure 1). In 2011, it reached an isolated salt works based in a natural lake near Lake Alexandrina, SA (M. Coleman, pers. comm.). This recent spread of parthenogenetic *Artemia* is not being monitored, and the public is not being made aware of the problem. The prediction that *A. franciscana* is likely to spread from salt works [9] has not yet been realised. Certainly it is persisting in places where salt used to be harvested such as near Port Augusta, SA, Hutt Lagoon and Koorkoodine WA, but it is not known to have spread from these either [10].

Most information on *Artemia* (e.g. [1, 11]) comes from the northern hemisphere, though some studies include comparative data from Australia (e.g. [12–14]). Although the large salt works in northwest Australia monitor *Artemia* activity (M. Coleman, pers. comm.), there has been no published research. Before 1975, little was known on *Artemia* and *Parartemia* in Australia, which Geddes & Williams tried to redress [6, 15–17] with comparisons of *Artemia* and the local *Parartemia*, but at the time *Artemia* was not spreading and the high biodiversity and physiology of *Parartemia* were unknown.

### *Parartemia*: taxonomy

It was not until 1903 that the first species of *Parartemia* (as *P. zietziana*, Sayce) was described [18]; any brine shrimp encountered before this date was thought to be *Artemia* (see above). In 1910, Daday [19] placed it in its own family, but it then spent more than 90 years in the family Branchipodidae until Weekers *et al*. [20], using molecular techniques, showed it to be a family in its own right, which vicariates with the Artemiidae and indeed is its sister group.

Presently, 18 described species of *Parartemia* are known [21–24] with at least one undescribed species [25]. Molecular analyses of most of these support specific delineations based on morphological differences, though *P. contracta* could represent two species [26], I. Kappas, pers. comm.). The separation of *P minuta* and *P. mouritzi*[24] from the remainder was also supported molecularly (I. Kappas, pers. comm). The great majority of *Parartemia* species occur in southwestern Western Australia: 13 occur in WA, 7 in SA (two of these shared with WA), 2 in Victoria and one each in NT, Queensland and Tasmania [all of latter four occurrences shared with SA or WA (Table 1)]. The high species richness in WA is thought to be associated with the abundance and diversity of saline habitats there and their great age, while contrastingly the low diversity in eastern Australia is in tune with the simplicity of saline environments there [6]. Also promoting diversity in WA is past climate changes [27] and the alleged poor dispersal abilities of *Parartemia* due to their eggs sinking and being bound to bottom substrates [10, 15]. Detailed distribution maps are available in Timms *et al*. [10] and Timms [24].

**Table 1:** **List of species of** ***Parartemia*** **and their gross distribution (from** [23] **)**

Species	Distribution
*P. acidiphila* [23]	Esperance hinterland and northeast Eyre Peninsula
*P. auriciforma* [23]	Inland northwest SA
*P. boomeranga* [45]	Inner northwestern Wheatbelt, WA
*P. bicorna* [45]	Lake Carey in northern Goldfields, WA
*P. contracta* [21]	Northern, central and southern Wheatbelt, WA
*P. cylindifera* [21]	Southern Wheatbelt WA, southern SA
*P. extracta* [21]	Northern and central Wheatbelt, WA
*P. informis* [21]	Northern and central Wheatbelt, WA
*P. laticaudata* [45]	Northwest and central inland WA, southwest NT
*P. longicaudata* [21]	Whole Wheatbelt, including Esperance, WA
*P. minuta* [44]	Inland Qld, western NSW, northeast SA, nw Vic
*P. mouritzi* [45]	Eastern Wheatbelt WA
*P. purpurea* [45]	Esperance hinterland, WA
*P. serventyi* [21]	Eastern Wheatbelt, southern Goldfields, WA
*P. triquetra* [23]	Remote northwest SA
*P. veronicae* [45]	Goldfields, WA
*P. yarleensis* [23]	Central SA
*P. zietziana* [18]	Southern SA, north and west Vic, central Tas
*Parartemia* species “e”	Lake Barlee, WA

**Figure 2:**
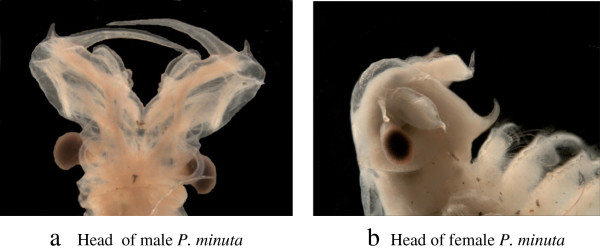
***Parartemia minuta*** **(a) head of male, (b) head of female.**

*Parartemia* males are distinctive in having the basal third of the second antenna fused (Figure 2a), which has a pair of dorsoventrally flattened rectangular or short blade-like distal processes and also a pair of digitiform anterior processes [24]. The distal segment of the antenna is long and slender and only slightly curving medially. Females (Figure 2b) are distinctive with an acute labrum pointing anteriodorsally (and hence easily distinguished from *Artemia* females) [23]. Most have various adaptations of the pregenital segments of the thorax (segments 8–11 in most, but as forward as segment 5 in *P. minuta*) in order to be clasped properly by the male antenna of the appropriate species [28]. Most females have brood chambers with lateral lobes though a few species have compact rounded chambers (both structures quite different from other Australian anostracans except *Artemia*) [24].

All species use a lock and key mechanism in amplexus [28] with the male distal antennomeres locking around the female 11^th^ thoracic segment or thereabouts and the anterior processes touching the female dorsa of thoracic segments 10 and 9 and maybe more depending on whether female segments are shortened by contraction and the length the male anterior processes. The female 11^th^ thoracopod is greatly reduced to facilitate this coupling. Keys to *Parartemia* species utilize these secondary sex characteristics [24].

In a sense it is a puzzle why *Parartemia* utilizes lock and key amplexus when congeneric occurrences are rare, so the need for preventing hybridisation is almost nil. However many species often occur in adjacent lakes in WA and SA, and it is possible eggs may perhaps disperse outside their own lake into a nearby lake with a different species. In this case, competitive exclusion would be served by the incumbent species being unable to mate in the first instance without wasting scarce resources (see below) on unfit hybrids.

### *Parartemia*: physiology

**Figure 3:**
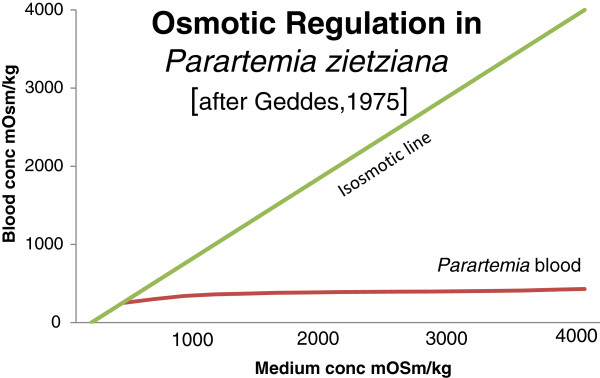
**Osmotic regulation in** ***Parartemia zietziana*** **(redrawn from [** [28] **]).**

*Parartemia zietziana* is an efficient hypo-osmotic regulator [29] (Figure 3). Like *Artemia,* it is a respiratory regulator, meaning the respiration rate remains constant over a wide range of oxygen concentrations. For *Parartemia*, regulation ceases at about 2 mg L^-1^ of oxygen, whereas for *Artemia* it stops at about 1 mg L^-1^ because of their ability to produce haemoglobin [30]. Osmoregulation accounts for only 3% of the total variance in respiratory rate [31].

*Parartemia zietziana* achieves regulation by sodium excretion via the Na, K-ATPase system [32]. This creates a heavy demand on cellular reserves of ATP, which is satisfied from increased glycogen breakdown with the ATP supplied by a facultative aerobic glycolytic cycle. The CO_2_ to run the C-4 pathway comes from dissolved carbonate and bicarbonate in alkaline saline waters plus some endogenous CO_2_ too [33]. This species has the highest known field salinity range, possibly because it has been more intensely studied. Other species known to inhabit hypersaline lakes >200 mg L^-1^ include *P. acidiphila, P. contracta, P. longicaudata, P. minuta, P. purpurea, P. serventyi* and *P. veronicae* (Table 2). Further field data on these and other species will no doubt raise their upper known limits, but probably not for at least *P. cylindifera*. In the Esperance hinterland and on Eyre Peninsula where series of lakes have been studied [34, 35] this species occurs in lower salinity lakes while others (*P. acidiphila, P. longicaudata, P. purpurea, P. zietziana*) live at higher salinities. *Parartemia extracta* may be another example of a species living at moderate salinities, this time in the northern Wheatbelt (A. Pinder, pers. comm.).

**Table 2:** **Lengths and salinity tolerance of** ***Parartemia*** **(from** [10] **)**

Seasonal lakes in Southern Australia			Episodic lakes in Inland Australia		
Species	Male length (mm)	Salinity tolerance (g L ^-1^ )	Species	Male length (mm)	Salinity tolerance (g L ^-1^ )
*P. acidiphila*	12.1	35–210	*P. auriciforma*	11.5	?
*P. boomeranga*	22.2	50–120	*P. bicorna*	18.4	22–105
*P. contracta*	20.1	84–240	*P. laticaudata*	17.5	8–141
*P. cylindifera*	22.3	3–123	*P. minuta*	14.2	2–225
*P. extracta*	17.0	27–100	*P. mouritzi*	10.5	33–95
*P. informis*	26.7	30–186	*P. triquetra*	19.5	?
*P. longicaudata*	25.4	41–240	*P. veronicae*	13.6	74–225
*P. purpurea*	21.5	20–235	*P. yarleensis*	18.0	?
*P. serventyi*	21.2	15–262			
*P. zietziana*	19.3	27–353			

In the Dry Creek Saltfield near Adelaide, *P. zietziana* and parthenogenetic *Artemia* (recorded as *A. salina* in the study) co-occur; *P. zietziana* alone at 112–214 g L^-1^ and *Artemia* alone above 285 g L^-1^ while both in the same ponds between 214 and 285 g L^-1^[30]. While *P. zietziana* can osmoregulate above 285 g L^-1^, it is at a disadvantage compared to *Artemia* because it lacks haemoglobin (see above).

Most species of *Parartemia* live in alkaline waters, but three species, *P. acidiphila, P. contracta, P. mouritzi*, naturally live in acid waters, where carbonate and bicarbonate are not available. Conte & Geddes [32] showed that *P. contracta* must have evolved an additional proton pump to produce ATP from endogenous CO_2_ and perhaps also from dissolved CO_2_. Presumably, *P. acidiphila* living as it does in waters down to pH 3.5 [34] is similarly endowed, but *P. mouritzi* may not be, as so far it has not been found below pH 6.2. *Parartemia serventyi*, though typically living in alkaline waters, can survive mildly acid conditions of secondary salinised waters (G. Janicke, pers. comm.), so perhaps it has similar physiological modifications. Survival mechanisms in acidified waters would obviously be a productive area for future investigations.

### *Parartemia*: aquaculture

*Artemia* is cultured worldwide and also in Australia, and in the early 2000s attempts were made to culture *Parartemia* commercially. Securing eggs was a problem, as they sink and become secure in the bottom substrates, so that the ready collection of floating eggs as in *Artemia* was impossible. The larger species were tried but unsuccessfully as growth rates were low and erratic (A. Savage, pers. comm.). Perhaps if the smaller species of the remote inland (see below) were tried there may have been some success, but egg collection remains a problem.

### *Parartemia*: ecology

In Victorian seasonal salinas, *Parartemia zietziana* hatches at 20–40 g L^-1^ as the sites fill in late autumn-winter, passes through 2–3 cohorts in late winter-spring and then dies as the salinity rises rapidly in late spring-early summer [36, 37]. Much the same probably applies to similar seasonal salinas in southern SA and WA but involving various species according to lake type and locality [34, 35, 38]. In the central inland, salt lakes fill episodically often in summer, and in the Paroo, *P. minuta* hatches at low salinities, grows and reproduces, passing through one or more generations and persists as long as salinities do not exceed its upper tolerance limit [3, 39].

*Parartemia zietziana* eats mainly organic matter stirred from the bottom [31] and it is assumed other species feed in the same way. If so, it explains why there is rarely more than one species per lake, as there is no opportunity for size selection of particles and hence partitioning the resource as in the case of *Branchinella* feeding on diatoms [40].

The various species of *Parartemia* exhibit a range of characteristic lengths (Table 2). In a superficial assessment not accounting for factors such as food supply, growth temperature and salinity (in high salinities less energy is available for growth, see below), species occurring in the more reliably filling and longer lasting salinas in southern Australia generally are larger than those occurring in the short hydroperiods of episodic lakes of the inland [10]. How this is mediated is unknown, but it ensures species of the inland can mature early and reproduce before the site dries.

Marchant [37] in studying the energetics of *P. zietziana* in two lakes near Colac, Victoria (Pink: 80–240 g L^-1^; Cundare: 50 g L^-1^ to dry) assimilation was only 1631 kJ m^-2^ yr^-1^ in Pink Lake and 212 kJ m^-2^ yr^-1^ in Cundare. Pink Lake is of very low productivity [41] and Cundare probably even lower; allochthonous inputs appear to be the main source of energy [42]. In both lakes, on average, respiration accounted for 60–80% of assimilation. However respiration was often greater than assimilation and this imbalance lead to continuous mortality, variable growth and unpredictable recruitment. Gross growth efficiency (i.e. production/assimilation) of 15–30% was in the same range for other anostracans, but net growth efficiency (i.e. production/consumption) of 5–12% was well reduced. Although *Parartemia* is well adapted to live in salt lakes, life is difficult.

### Halophilic *Branchinella*

While generally not being called brine shrimps, a few species of the freshwater genus *Branchinella* live in saline waters. Most notable among these is *Branchinella simplex* in salinas in the middle Goldfields of WA and *B. buchananensis* in some saline lakes in northwest NSW and inland Qld (Figure 1). The upper field salinity limit for *B. simplex* is 62 g L^-1^ and for *B. buchananensis* is 42 g L^-1^[3]. Other species have much lower upper limits: *B. compacta* 15.9 g L^-1^, *B. papillata* 14 g L^-1^, *B. nana* 13 g L^-1^, *B. nichollsi* 12 g L^-1^, *B. erosa* and *B. hearnii* 12 g L^-1^, *B. australiensis* 11.2 g L^-1^, *B. affinis* 6.7 g L^-1^ and *B. frondosa* 4.2 g L^-1^ ([3], unpublished data). The occurrence of these species of *Branchinella* in saline lakes is no accident and is part of the normal succession of species in the lakes in which they occur, e.g. *B. buchananensis* in Lake Gidgee, the Paroo [3], *Branchinella hearnii* in the Unicup lakes near Manjimup, southwest WA (R. Hearn, pers. comm.), *B. nana*, *B. nichollsi* and *B. simplex* in Arrow Lake near Kalgoorlie [43] and *B. simplex* in Lake Carey, WA (B. Datson, pers. comm.). In some cases as the Unicup series there is no local *Parartemia* adapted to hyposaline waters and the waters do not exceed low salinities but in other cases such as in Gidgee Lake and Lake Carey the lake eventually becomes hypersaline and *Parartemia* assumes dominance. There is probably no competition between *Branchinella* and *Parartemia* as each eats different foods (see above) and it is purely a matter of salinity tolerance determining which is dominant.

Geddes [44] showed *B. australiensis* and *B. compacta* to be osmoconformers and presumably this applies to other species of *Branchinella* living in hyposaline waters. However it would be remarkable if this was the survival mechanism for *B. buchananensis* and *B. simplex* in mesosaline and hypersaline waters, respectively. To date the episodic occurrence of these species in remote salinas has mitigated against the solving of their osmoregulatory mechanism.

Four of the hyposaline *Branchinella, B. australiensis, B. compacta, B. erosa* and *B. hearnii*, have modifications to their posterior thorax, possibly to enhance species recognition by the male or contribute to lock and key mating [45]. Why these modifications should be almost confined to hyposaline species (one supposedly freshwater species, *B. vosperi*, has the posterior thorax modified) is unknown, but three (not *B. hearnii*) may come in contact with *Parartemia* which employs the lock and key mechanism in amplexus (see above), so they would be unable to connect in mating. In that *B. vosperi* was found within 100 m of hyposaline lakes with *Parartemia*, it is possible it also occurs in such lakes, but it was missed in totally inadequate collecting (author, unpublished data). By contrast, all freshwater species (except *B. vosperi*), despite common congeneric occurrences, lack female thoracic modifications, as according to this theory they never meet *Parartemia*.

### Brine shrimps: conservation

A general perception is *Parartemia* spp. live in remote salt lakes well removed from human influences on the environment. This is not the case in the Western District of Victoria, Eyre Peninsula SA or the Wheatbelt of WA, where extensive land clearing is associated with grain farming, dairying or grazing. Also, some remote inland salt lakes are impacted by mining in WA.

In Victorian agricultural regions no change has been noted in the only species present, *P. zietziana*, possibly because its wide salinity tolerance masks changes in lake salinities. Certainly it now lives in Lake Corangamite where previous mesosaline conditions enabled predatory fish to survive. This change has been largely due to diversion of the main inflowing stream and hence an increase in salinity by evaporation [46]. On Eyre Peninsula, SA, lakes are salinising and Timms [35] predicts *P. cylindifera* will be replaced by the more tolerant *P. zietziana*. In WA there is extensive salinisation of lakes, so that populations of *Parartemia* spp. have been lost in many lakes [10]. The driving factors for this loss are not so much salinity increase, but associated acidification and changed hydrology from temporary seasonal to permanent waters [10]. Also in WA, there are a few lakes in the Goldfields where saline groundwater is discharged from mining activity. Despite concerns this may be inimical to hyposaline communities [46], this is not the case at least in Lake Carey (M. Coleman, pers. comm.).

At the species level, there are concerns for five species. *Parartemia boomeranga* naturally occurred over a small area, and it seems all the lakes in this area are now too salinised to support it, while *P. extracta’s* distribution has shrunk as lakes in its domain salinised [10]. These authors suggest both should be given an IUCN Vulnerable Status, but this has not been acted upon because of lack of sufficient evidence. In an effort to get such data for *P. extracta*, it seems it is safe in some core lakes in its distribution (A. Pinder, pers. comm.) and its known distribution has actually been expanded (author, unpublished data).

With just four known sites for *P. mouritzi*, two of which are mildly salinised, Timms *et al*. [10] suggested this be given local classification of WA DPAW Priority 1 species. Similarly, *P. bicorna* and the undescribed *Parartemia* sp. “e” occur in just one lake each, and a DPAW Priority 4 allocation is appropriate. These suggestions have not been acted upon because authorities want extensive field collections to prove these cases. This is difficult for extremely episodic and remote lakes. For instance, recently *P. bicorna* has been found in Lake Minigal (B. Datson, pers. comm) and Lake Ballard (R. Pedler, pers. comm.), both of which had filled for the first time in years and a collector had visited at a propitious time.

No recommendations have been made for species of *Parartemia* in other states, though they are considered safe enough in settled areas (Qld, NSW, Vic, Tas, SA) or their known occurrences are so remote (*P. auriciforma, P. triquetra* in SA, *P. laticaudata* in NT) that there are no concerns. The salt tolerant fairy shrimp, *Branchinella buchananensis* is considered endangered in New South Wales and under the Fisheries Management Act of 1994 it has been declared a vulnerable fish (!) species. It occurs in a few [47] known localities in the state and one of these contains an economic deposit of gypsum, which in the 1990s was proposed to be mined. The present owners of the lake are much more sympathetic to its ecology.

## Conclusions

Australia has three genera and 32 species of anostracans in its saline lakes. The otherwise freshwater *Branchinella* is largely restricted to hyposaline waters. Most of its species probably osmoconform, but two at higher salinites may not. Native parthenogenetic *Artemia* is spreading into altered salinas, but introduced *A. franciscana* is not, despite predictions. *Parartemia* (19 species) generally hypo-osmoregulate almost as competently as *Artemia* but are restricted at very high salinities as they lack haemoglobin to function in the low oxygen environments. Populations can suffer continuous mortality through lack of food resources.

Mating in *Parartemia* is by lock and key amplexis, but why this should be so is not easily explained, given species almost never co-occur. Species are smaller where salinas have shorter hydrologies, perhaps an adaptation to increase the chances of maturation. The ecology of only one species has been extensively investigated; others may live differently, especially those in acid saline waters, an obvious gap in knowledge.

Many saline lakes in Australia are being modified, particularly in the Wheatbelt of WA, so that some species are at risk. Except for a species in NSW which has been designated a vulnerable fish (!), none has been protected, largely because authorities require more data and such are hard to get for species living in remote episodic sites.

## Abbreviations

NSW: New South Wales
NT: Northern Territory
Qld: Queensland
SA: South Australia
Tas: Tasmania
WA: Western Australia
IUCN: International Union for Conservation of Nature
DPAW: Department of Parks and Wildlife.
